# Enantioselective Separation of Mirtazapine and Its Metabolites by Capillary Electrophoresis with Acetonitrile Field-Amplified Sample Stacking and Its Application

**DOI:** 10.3390/molecules19044907

**Published:** 2014-04-17

**Authors:** Jun Wen, Wen-Ting Zhang, Wei-Qun Cao, Ji Li, Fang-Yuan Gao, Nan Yang, Guo-Rong Fan

**Affiliations:** 1 Department of Pharmaceutical Analysis, School of Pharmacy, Shanghai Key Laboratory for Pharmaceutical Metabolite Research, Second Military Medical University, Shanghai, 200433, China; E-Mails: wenjunapple@163.com (J.W.); zhangwt2011@163.com (W.-T.Z.); cao_weiqun@wuxiapptec.com (W.-Q.C.); gaofangyuan@outlook.com (F.-Y.G.); luckyyangnan123@163.com (N.Y.); 2 Department of Pharmacy, Kunming General Hospital of Chengdu Military Command, Kunming, 650032, China; E-Mail: gigiliji@163.com

**Keywords:** chiral capillary zone electrophoresis, acetonitrile-field-amplified sample stacking, mirtazapine, N-demethylmirtazapine, 8-hydroxymirtazapine, liquid-liquid extraction

## Abstract

A simple, rapid and sensitive chiral capillary zone electrophoresis coupled with acetonitrile-field-amplified sample stacking method was developed that allows the simultaneous enantioselective separation of the mirtazapine, N-demethylmirtazapine, 8-hydroxymirtazapine and mirtazapine-N-oxide. The separation was achieved on an uncoated 40.2 cm × 75 μM fused silica capillary with an applied voltage of 16 kV. The electrophoretic analyses were carried out in 6.25 mM borate–25 mM phosphate solution at pH 2.8 containing 5.5 mg/mL carboxymethyl-β-cyclodextrin. The detection wavelength was 200 nm. Under these optimized conditions, satisfactory chiral separations of four pair enantiomers were achieved in less than 7 min *in vitro*. After one step clean-up liquid-liquid extraction using 96-well format, sample was introduced capillary zone electrophoresis with acetonitrile-field-amplified sample stacking to enhance the sensitivity of enantiomers. The method was validated with respect to specificity, linearity, lower limit of quantitation, accuracy, precision, extraction recovery and stability. The lower limit of quantification was 0.5 ng/mL with linear response over the 0.5–50 ng/mL concentration range for each mirtazapine, N-demethylmirtazapine and 8-hydroxymirtazapine enantiomer. The developed and validated method has been successfully applied to the enantioselective pharmacokinetic studies in 12 healthy volunteers after oral administration of rac- mirtazapine.

## 1. Introduction

**Figure 1:**
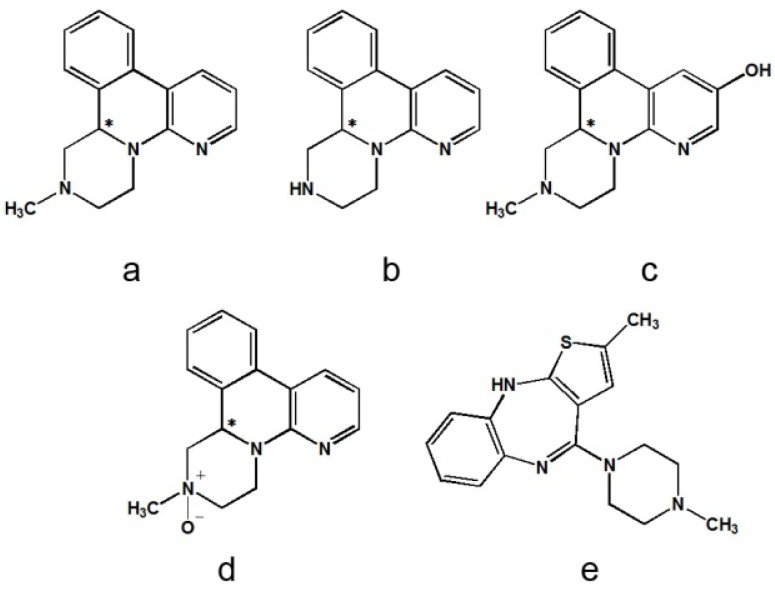
Chemical structures of (**a**) mirtazapine (MRT); (**b**) demethylmirtazapine (DMR); (**c**) 8-hydroxymirtazapine (8-OHM); (**d**) mirtazapine-N-oxide (N-O-MRT); (**e**) olanzapine (IS); (*) denotes a chiral center.

Mirtazapine (MRT), whose chemical name is 1,2,3,4,10,14b-hexahydro-2-methylpyrazino[2,1-a] pyrido[2,3-c][2]benzazepine (Figure 1), is an effective tricyclic antidepressant combining two mechanisms of action: antagonizing the adrenergic a_2_-autoreceptors and a_2_-heteroreceptors as well as blocking postsynaptic serotonin 5-HT_2_ and 5-HT_3_ receptors. It also shows low affinity for 5-HT_1A_ receptors and therefore could enhance the release of norepinephrine and 5-HT_1A_–mediated serotonergic transmission. Thus, it is classified as a noradrenergic and specific serotonergic antidepressant (named as a NaSSA) [1,2,3,4,5]. It is also has been introduced in the veterinary field as an attractive ingredient to reduce pain, vomiting and to increase appetite in cats, dogs and horses [6,7,8,9]. Currently, MRT is marketed as the racemate and its (−)-*R* and (+)-*S*-enantiomers have different pharmacologic and pharmacokinetic properties. The (+)-*S*-enantiomer shows a greater binding affinity than (−)-*R*-MRT in blocking a_2_-adrenergic auto receptor sand 5-HT_2_, whereas the a_2_-adrenergic hetero receptor and 5-HT_3_ type receptor blockading potencies reside predominantly in the (−)-*R*-enantiomer. In addition, the enantioselective pharmacokinetics of MRT indicated the difference in half-life of the enantiomers. The elimination rate of the (+)-*S*-MRT was larger than that of the (−)-*R*-MRT after oral administration of the racemate [10,11]. 

Major pathways of biotransformation of MRT were demethylation with the formation of N-demethylmirtazapine (DMR) and hydroxylation with the formation of 8-hydroxymirtazapine (8-OHM) followed by conjugation in humans. N-oxide and N^+^-glucuronide of MRT were also found in plasma and urine after oral administration of MRT [12]. *In vitro* study demonstrated that enantioselective preference was shown by three cytochrome P450 enzymes, whereby (+)-*S*-MRT was metabolized extensively by CYP2D6 and to a relatively smaller extent by CYP1A2, and also to a relatively small extent (−)-*R*-MRT was metabolized by CYP3A4 [13]. 

Several analytical separation techniques had been reported for chiral separations of MRT alone or together with the metabolites in various biosamples, such as HPLC-UV [14,15,16,17], LC-FLD [18] and LC–MS/MS [19,20,21] on chiral column with cellulose or vancomycin as chiral stationary phase. In order to preconcentration and cleanup the biological fluids for simultaneous analysis of enantiomers, traditional sample preparation techniques including liquid-liquid extraction (LLE) [16,19], solid-phase extraction (SPE) [15,17] and protein precipitation [18] were used as well as some relative new techniques including liquid-phase microextraction (LPME) [14,21] and solid-phase microextraction (SPME) [20]. However the major drawback of these methods was the long analysis time (> 15 min) except for a LC–MS/MS determination within 10 min described by de Santana [21]. In addition the sample preparation procedures were not only tedious and time consuming but also use larger volume of plasma or urine except protein precipitation. 

On the other hand, capillary electrophoresis (CE) has become a promising, effective and economic approach in the field of chiral separation of drugs with various separation modes. It has several advantages compared to those techniques mentioned above, including high efficiency, rapid separation, low consumption of sample and separation electrolytes, and far cheaper capillaries. Hence CE method with carboxymethyl-β-cyclodextrin (CM-β-CD) as chiral selectors [22,23] and capillary electrochromatography with vancomycin as chiral solid phase [24] were employed for the enantioselective separation of MRT and its metabolites. Mandrioli *et al.* [22] first reported a CE method to enantioseparate MRT and DMR in human plasma within a very brief time with a lower limit of quantification (LLOQ) of 7.5 ng/mL. Of late, Malagueno *et al.* [23] have published a validated CE chiral method for the simultaneous analysis of the enantiomers of MRT, 8-OHM and DMR in human urine within 18 min with a limit of quantification (LOQ) of 62.5 ng/mL. To overcome the drawback of the sensitivity in determination of the low concentration of the analytes in human plasma or urine by CE method, SPE [22,24] or LPME [23] was also developed for the purification and concentration of the biological samples by using high biological fluids volume. 

An alternative approach to overcome the limit of sensitivity in CE was field amplified sample stacking (FASS) which was considered as a simple and efficient technique in capillary zone electrophoresis (CZE), first introduced by Mikkers *et al.* [25]. Shihabi [26] reported that organic solvent field amplified yields far better stacking than aqueous solvent field amplified for the compounds. Recently, our group has used acetonitrile field amplified sample stacking (ACN-FASS) technique for online concentration in CZE for the analysis some drugs in plasma [27,28]. Samples were dissolved in acetonitrile-water and then electrokinetically injected into the capillary. The conductivity of the samples was much lower than that of the background electrolyte (BGE) solution, resulting in enhanced electric field strength at the injection end. The analytes in the sample zone with high migration speed would decelerate sharply at the boundary of the running buffer, and therefore condensed at the interface between the low-conductivity zone and the running buffer [26,29]. The approach could effectively improve the sensitivity about 50–60 folds.

In this paper, we developed a CE method for the simultaneous enantioselective analysis of MRT and its three metabolites *in vitro* combined with ACN-FASS. Several parameters, such as type and concentration of chiral additives, pH and concentration of the BGE and applied voltage were studied for the rapid and sensitive chiral analysis optimization. One step plasma sample preparation by LLE in 96-well format was implemented to increase the throughput. After the sample preparation, N-O-MRT was not extracted from the plasma, which was coincided with the published article [19]. Thus, the method was validated for the simultaneous determination of MRT, DMR and 8-OHM enantiomers in human plasma, which offers lower LLOQ (0.5 ng/mL) with 200 μL plasma for each enantiomers and short run time (7 min). The present method was applied to the enantioselective pharmacokinetic studies in 12 healthy volunteers after oral administration of *rac*-MRT.

## 2. Results and Discussion

### 2.1. Optimization of Enantioseparation Conditions

#### 2.1.1. CD Type and Concentration

The chiral separation in CE by the use of CDs utilizes the phenomenon of host-guest complexation with a relatively hydrophobic cavity, where a transient diastereomeric complex is formed between the chiral selector and the analytes [30]. In order to obtain simultaneous baseline resolution of four pair enantiomers, MRT, DMR, 8-OHM and N-O-MRT, β-CD and its derivatives were studied in our experiments as chiral selector according to the published papers [22,23]. Suitable chiral resolution was observed when using CM-β-CD, which coincided with these two papers.

**Figure 2:**
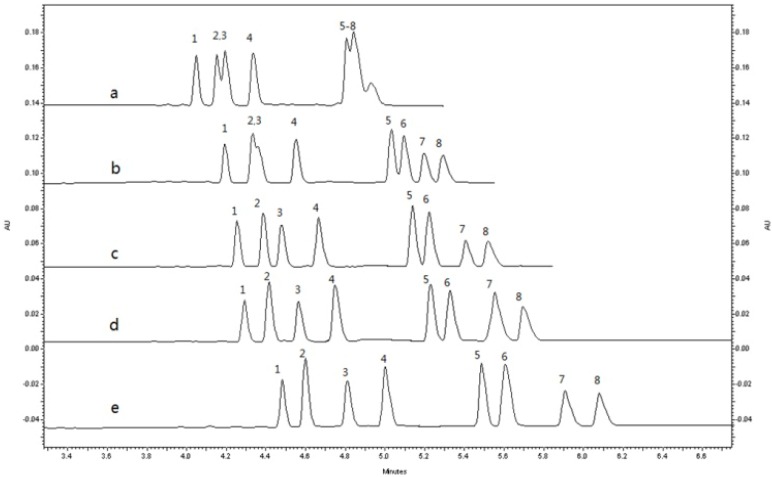
Electropherograms of *rac*-MRT (200 ng/mL) and its metabolites standard with different chiral selectors (CM-β-CD) concentrations in the BGE: (**a**) 3.0 mg/mL; (**b**) 4.0 mg/mL; (**c**) 4.5 mg/mL; (**d**) 5.0 mg/mL; (**e**) 5.5 mg/mL. Sample solvent: 10% buffer solution; Experimental conditions: running buffer: 25mM phosphate–6.25mM borate (pH3.0); total uncoated capillary length: 40.2 cm × 75 μM i.d, effective length: 30.2 cm; applied voltage:16 kV (+)→(−); column temperature: 20 °C; detection wavelength: 200 nm; electrokinetic injection: 7.5 kV × 10 s. Peaks: (1) (−)-*R*-DMR; (2) (−)-*R*-MRT; (3) (+)-*S*-DMR; (4) (+)-*S*-MRT; (5) (−)-*R-*8-OHM; (6) (+)-*S*-8-OHM; (7) (−)-*R*-N-O-MRT; (8) (+)-*S*-N-O-MRT.

As shown in Figure 2, with increasing CM-β-CD concentrations from 3.0 to 5.5 mg/mL, the migration time was prolonged. At the concentration of 5.5 mg/mL, the resolution between pairs of enantiomers of *rac*-8-OHM, the most difficult to be separate, was improved and the reproducibility was better. When the concentration of CM-β-CD was further increased, the resolution was slightly increased and the analysis time was longer. So the concentration of 5.5 mg/mL was selected as the optimal concentration.

#### 2.1.2. Optimization of Borate-Phosphate Buffer pH and Concentration and Applied Voltage

**Figure 3:**
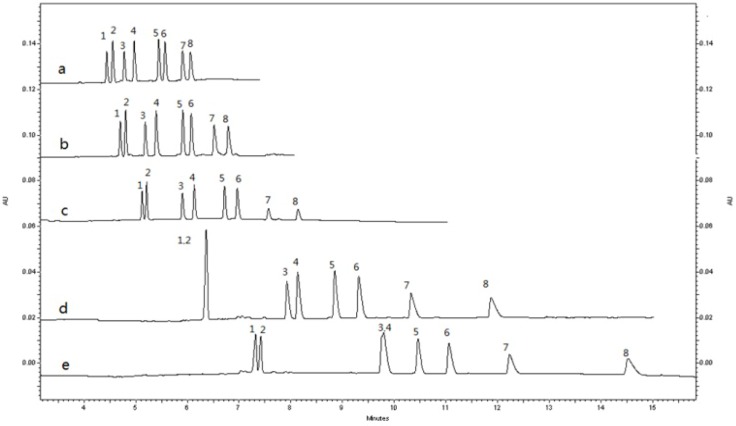
Electropherograms of *rac*-MRT(200 ng/mL) and its metabolites standard with different buffer pH: (**a**) pH 2.8; (**b**) pH 3.0; (**c**) pH 3.2; (**d**) pH 3.4; (**e**) pH 3.6. Peaks: (1) (−)-*R*-DMR; (2) (−)-*R*-MRT; (3) (+)-*S*-DMR; (4) (+)-*S*-MRT; (5) (−)-*R*-8-OHM; (6) (+)-*S*-8-OHM; (7) (−)-*R*-N-O-MRT; (8) (+)-*S*-N-O-MRT. Other conditions see Figure 2.

MRT and its metabolites are basic drugs and they can be fully protonated in buffer at acidic pH values. Figure 3 showed that running buffer containing 6.25 mM borate–25 mM phosphate solution and 5.5 mg/mL CM-β-CD was studied at different pH value. The increase of the pH from 2.8 to 3.6 prolonged the migration time and worsened the resolution. With a pH of 2.8, better resolution and run time were obtained (Figure 3).

Different buffer solutions had different effects on the separation performance through its influence on the EOF and the current produced in the capillary [31]. The effect of concentration of borate-phosphate buffer (pH 2.8) was studied. With the increasing buffer concentration, improved peak shape and resolution was obtained. 

However, higher concentration might generate the increase in current and lower concentration meant lower conductivity resulted in loss in separation and sample stacking. Finally, 6.25 mM borate–25 mM phosphate buffer solution was employed to achieve a good compromise between resolution, sensitivity and analysis time. Voltages over the range from 12 to 20 kV were investigated. Too large of a separation voltage induced certain current problems, such as appreciable Joule heating and 16 kV was chosen because it provided better performance.

#### 2.1.3. Optimization of Sample Stacking Performance

#### 2.1.3.1. Selection of Injection Modes, Time and Voltage

The analytes having higher pKa than the pH of the sample solution can be efficiently injected at positive polarity with electrokinetic injection [32] and sample stacking with electrokinetic injection provide larger sensitivity enhancements compared with hydrodynamic injection [33]. Therefore the electrokinetic injection was chosen as the optimum for the analysis of basic drugs in this experiment.

Electrokinetic injection sampling was used with solutes dissolved in 10% buffer (6.25 mM borate–25 mM phosphate ), and the injection time (5 s–30 s) was tested with the injection voltage set at 3 kV. With increasing the injection time, the sensitivity was enhanced. Because of the overload of the sample with longer injection time, the response of analytes was reduced when the injection time was >20 s. So the injection time was set at 20 s. With the 20 s injection time, different injection voltages (3 kV–10 kV) were studied and the similar results mentioned above was observed. The low conductivity of sample solution and high injection voltage might cause the current break down, which would lead to the failure of injection. As a compromise, 7.5 kV was selected as the injection voltage.

#### 2.1.3.2. Selection of Sample Solvent

**Figure 4:**
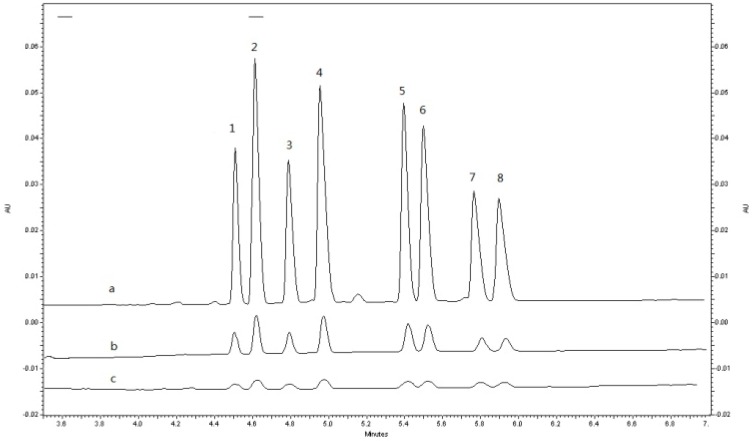
The effect of different sample solvents on FASS of *rac*-MRT (50 ng/mL)and its metabolites standard. Running buffer: 25 mM phosphate-6.25 Mm borate (pH 2.8) with 5.5 mg/mL CM-β-CD; Electropherograms: (**a**) 80% ACN–distilled water; (**b**) distilled water; (**c**) 10% buffer. Peaks: (1) (−)-*R*-DMR; (2) (−)-*R*-MRT; (3) (+)-*S*-DMR; (4) (+)-*S*-MRT; (5) (−)-*R*-8-OHM; (6) (+)-*S*-8-OHM; (7) (−)-*R*-N-O-MRT; (8) (+)-*S*-N-O-MRT. Other conditions see Figure 2.

The mechanism of ACN-FASS was creating a highly different fields strength between the sample zone and the separation electrolyte, which causes the analyte (cations) to experience high field strength, migrate rapidly and stack as a sharp band at the boundary buffer so as to bring along a focusing effect [34,35]. Increasing the difference of conductivity between sample solution and BGE could enhance the performance of sample stacking. Electropherograms for enantioseparation obtained after ACN-FASS are shown in Figure 4. There is about 50–60 times on the average increase in peak height due to the presence of ACN in the sample, which is similar to the results of some published papers [22,23,24]. Different ratios of ACN to distilled water (70%, 80%, 90%) were also investigated. With the increase of ACN concentration, the analytes were stacked more effectively. However, 90% ACN produced unstably current in CZE analysis, we chose 80% ACN–distilled water as sample solvent. 

Figure 4a showed the typical electropherograms of enantioselective analysis of MRT and its three metabolites under the optimal conditions within 7 min. The determination of the elution order using pure enantiomers was impossible because the individual standards of *S*- and *R*-enantiomers of MRT and its metabolites were not commercially available. Thus, the migration order of *rac*-MRT and its metabolites were defined by some published papers that also used CM-β-CD as chiral additives in BGE [18,19]. Under the adopted experimental conditions, the (−)-*R*-isomer migrated first and the (+)-*S*-isomer was the second migrating peak both for the drug and the metabolites.

#### 2.1.4. Sample Preparation

According to the method described in references [16,19], one step LLE for the extraction of plasma samples was optimized in our preliminary studies. Ethyl acetate and methyl *tert*-butyl ether were tested as the extraction solvent. The latter was adopted because of its high extraction efficiency and efficient sample clean-up results. A 20 μL aliquot of 1 mol/L NaOH was added to 200 μL plasma spiked with 20 μL internal standard (IS) solution (200 ng/mL olanzapine), which avoided analytes protonation during extraction. In order to increase sample throughput, the LLE 96-well plates were used, resulting in a shorter sample preparation time.

### 2.2. Method Validation

#### 2.2.1. Specificity

**Figure 5:**
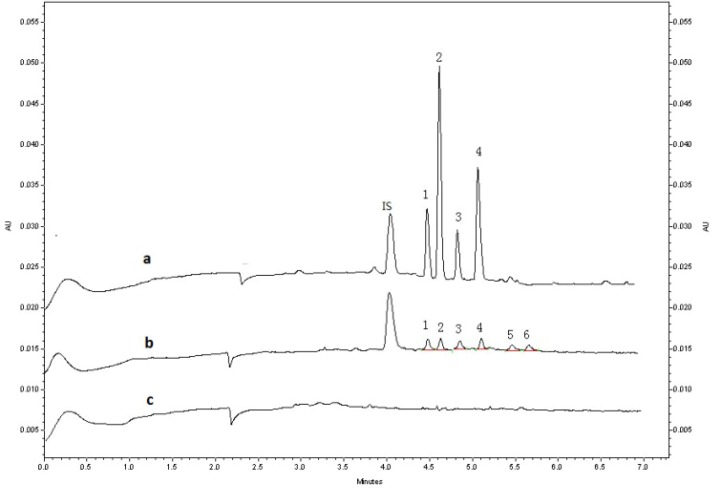
Typical electropherograms: (**a**) test sample of human treated with *rac*-MRT 0.5 h after the dose of 30 mg; (**b**) blank plasma spiked with 1ng/mL (LLOQ) *rac*-MRT, *rac*-DMR, *rac*-8-OHM and 20 ng/mL IS; (**c**) blank plasma. Peaks: (1) (−)-*R*-DMR; (2) (−)-*R*-MRT; (3) (+)-*S*-DMR; (4) (+)-*S*-MRT; (5) (−)-*R*-8-OHM; (6) (+)-*S*-8-OHM.

Specificity of the method was investigated by blank plasma, blank plasma spiked with *rac*-MRT, *rac*-DMR, *rac*-8-OHM at LLOQ of 1 ng/mL, and a real human plasma sample to discriminate the analyte from all potentially interfering substance. As shown in Figure 5, there was no significant interference from endogenous substances observed at the retention times of the analytes. The extraction procedure of plasma samples spiked 1 ng/mL and 100 ng/mL *rac*-N-O-MRT showed that N-O-MRT was not extracted, which was coincided with the published article [19] and the method was not further validated for the enantiomers N-O-MRT in human plasma. 

#### 2.2.2. Linearity of Calibration Curves and Lower Limits of Quantification

A series of different concentrations of 200 μL drug free plasma samples spiked with 1, 2, 5, 10, 20, 50 and 100 ng/mL of *rac*-MRT, *rac*-DMR and *rac*-8-OHM (n = 3) were analyzed to determine the linear response range. The calibration curves were generated by the peak area ratios of each enantiomer to IS *versus* the concentration of the half of the concentration of racemate spiked in the samples. A weighted (1/x) linear regression was used to perform standard calibration. Table 1 showed excellent linearity in the range 0.5–50 ng/mL of each MRT, DMR and 8-OHM enantiomer. The LLOQ of the assay was determined as the lowest concentration on the standard curve that could be quantitated with a precision of 20% and accuracy within ±20%. The LLOQ of MRT, DMR and 8-OHM enantiomers in human plasma was found to be 0.5 ng/mL with accuracy ranged from −10.8% to 10.4% and precision of 14% over five analytical runs.

#### 2.2.3. Accuracy, Precision and Extraction Recovery

Five replicate samples of quality control (QC) at three levels (low, medium, and high concentrations, 1, 5, 40 ng/mL) were analyzed in three separate runs. Precision was determined by calculating the coefficient of variation for within- and between-run replicates. Accuracy was assessed by calculating the percentage deviation from the spiked concentration. Table 2 and Table 3 showed that the within-day variances for all compounds were lower than 14.2% and all between-day variances were below 12.9%. It was shown that the within- and between-day accuracies were found to be within 10.4% and −10.8% for all the enantiomers

**Table 1:** The calibration equations for the determination of MRT, DMR, 8-OHM enantiomers (n = 3).

Enantiomer	Range(ng/mL)	Regression Equations	Correlation Coefficient (R^2^)	SD for the Slope	SD for the Inercept
(−)-*R*-MRT	0.5–50	y = 0.256x + 0.0188	0.998	0.050	0.027
(+)-*S*-MRT	0.5–50	y = 0.232x + 0.0156	0.992	0.048	0.015
(−)-*R*-DMR	0.5–50	y = 0.315x − 0.0314	0.995	0.037	0.030
(+)-*S*-DMR	0.5–50	y = 0.290x − 0.0290	0.993	0.041	0.020
(−)-*R*-8-OHM	0.5–50	y = 0.145x − 0.0517	0.995	0.044	0.030
(+)-*S*-8-OHM	0.5–50	y = 0.130x − 0.0444	0.994	0.039	0.033

**Table 2:** Within-day precision and accuracy for the analysis of MRT, DMR and 8-OHM enantiomers in human plasma (n = 5).

Enantiomer	LLOQ			Low QC			Medium QC			High QC		
	0.5 ng/mL			1 ng/mL			5 ng/mL			40 ng/mL		
	Mean ± SD	RE	RSD	Mean ± SD	RE	RSD	Mean ± SD	RE	RSD	Mean ± SD	RE	RSD
	(ng/mL)	(%)	(%)	(ng/mL)	(%)	(%)	(ng/mL)	(%)	(%)	(ng/mL)	(%)	(%)
(−)-*R*-MRT	0.54 ± 0.041	8.0	8.2	1.08 ± 0.12	7.8	11.3	5.16 ± 0.36	3.3	7.0	40.34 ± 1.4	0.8	3.4
(+)-*S*-MRT	0.46 ± 0.060	−8.1	14.0	0.99 ± 0.10	−1.2	10.0	4.88 ± 0.33	−2.4	6.7	39.32 ± 1.3	−1.7	3.4
(−)-*R*-DMR	0.55 ± 0.069	10.4	13.2	1.03 ± 0.15	2.7	14.2	4.61 ± 0.27	−7.9	6.0	39.57 ± 1.2	−1.1	3.0
(+)-*S*-DMR	0.49 ± 0.052	−2.2	9.2	1.04 ± 0.11	3.7	10.8	5.08 ± 0.25	1.6	4.9	39.28 ± 1.5	−1.8	3.9
(−)-*R*-8-OHM	0.45 ± 0.041	−10.8	7.9	0.97 ± 0.09	−3.4	9.7	4.68 ± 0.30	−6.3	6.3	43.68 ± 1.8	9.2	4.0
(+)-*S*-8-OHM	0.54 ± 0.041	7.6	8.2	0.95 ± 0.08	−4.8	8.8	5.35 ± 0.28	6.9	5.3	39.94 ± 0.95	−0.2	2.4

**Table 3:** Between-day precision and accuracy for the analysis of MRT, DMR and 8-OHM enantiomers in human plasma (n = 15).

Enantiomer	Low QC			Medium QC			High QC		
	1 ng/mL			5 ng/mL			40 ng/mL		
	Mean ± SD	RE	RSD	Mean ± SD	RE	RSD	Mean ± SD	RE	RSD
	(ng/mL)	(%)	(%)	(ng/mL)	(%)	(%)	(ng/mL)	(%)	(%)
(−)-*R*-MRT	1.04 ± 0.11	4.3	10.2	5.10 ± 0.36	2.1	7.1	41.26 ± 1.8	3.1	4.3
(+)-*S*-MRT	0.99 ± 0.10	1.0	9.5	4.98 ± 0.32	−0.5	6.5	39.69 ± 1.4	−0.8	3.6
(−)-*R*-DMR	1.07 ± 0.14	6.8	12.9	4.86 ± 0.33	−2.6	6.8	39.70 ± 1.3	−0.8	3.2
(+)-*S*-DMR	1.02 ± 0.10	1.6	9.6	5.05 ± 0.29	0.9	5.7	39.08 ± 1.5	−2.2	3.9
(−)-*R*-8-OHM	0.93 ± 0.10	−6.7	10.8	4.92 ± 0.35	−1.6	7.2	42.83 ± 1.8	7.1	4.1
(+)-*S*-8-OHM	0.99 ± 0.09	−0.6	9.0	5.14 ± 0.31	2.8	6.1	40.61 ± 1.5	1.5	3.6

The extraction recovery of MRT, DMR and 8-OHM enantiomers was determined by comparing the peak area of the QCs with the peak area of the corresponding standard solution spiked in extracted blank plasma. The recovery of IS was determined similarly. Table 4 showed the extraction recoveries were found to be all above 80% at three QC levels of MRT, DMR and 8-OHM enantiomers and the one of IS was 81.6%.

**Table 4:** Recoveries of MRT, DMR and 8-OHM enantiomers in human plasma (n = 5).

Enantiomer	Concentration (ng/mL)	Recovery (%) (mean ± SD)	RSD (%)
(−)-*R*-MRT	1	86.7 ± 8.9	10.3
	5	89.4 ± 5.7	6.4
	40	90.2 ± 4.8	5.3
(+)-*S*-MRT	1	87.7 ± 7.3	8.4
	5	90.1 ± 3.9	4.3
	40	92.0 ± 5.4	5.9
(−)-*R*-DMR	1	82.8 ± 8.6	10.4
	5	87.5 ± 7.6	8.7
	40	87.5 ± 3.0	3.4
(+)-*S*-DMR	1	83.5 ± 6.8	8.2
	5	85.8 ± 8.9	10.3
	40	88.9 ± 3.7	4.1
(+)-*R*-8-OHM	1	81.8 ± 5.9	7.3
	5	85.1 ± 8.7	10.2
	40	89.5 ± 2.3	2.5
(+)-*S*-8-OHM	1	83.2 ± 9.6	11.5
	5	86.1 ± 7.5	8.8
	40	87.8 ± 4.4	5.0

#### 2.2.4. Stability

Analyte stability determinations comprised short-term temperature stability, long-term stability, autosampler stability and freeze–thaw cycles stability, which were evaluated by analyzing three QC levels in quintuple. The peak areas obtained from both stability tests were compared with the peak areas obtained with freshly prepared samples. The mean values and standard deviations of the ratios between the concentrations found and initial concentration were used for stability evaluation. Table 5 showed that MRT, DMR and 8-OHM enantiomers in the spiked plasma samples were found to be stable in the experimental conditions assayed.

**Table 5:** Stability results of MRT, DMR and 8-OHM enantiomers in spiked plasma samples (n = 3).

Enantiomer	Nominal Concentration (ng/mL)	Freeze-Thaw Stability ^a^		30-Day Stability ^b^		Bench Top Stability ^c^		Autosampler Stability ^d^	
		Measured Concentration (ng/mL) (mean ± SD)	RSD (%)	Measured Concentration (ng/mL) (mean ± SD)	RSD (%)	Measured Concentration (ng/mL) (mean ± SD)	RSD (%)	Measured Concentration (ng/mL) (mean ± SD)	RSD (%)
(−)-*R*-MRT	1	0.99 ± 0.11	11.5	1.08 ± 0.12	10.8	1.04 ± 0.12	11.8	0.99 ± 0.10	9.7
	5	4.70 ± 0.25	5.4	4.98 ± 0.29	5.8	5.33 ± 0.29	5.5	5.13 ± 0.31	5.9
	40	37.61 ± 2.0	5.4	41.96 ± 1.3	3.0	41.33 ± 2.3	5.5	38.34 ± 1.4	3.6
(+)-*S*-MRT	1	0.95 ± 0.09	9.7	1.07 ± 0.12	1.6	1.02 ± 0.12	12.3	1.03 ± 0.12	11.8
	5	5.06 ± 0.35	6.9	4.89 ± 0.28	5.8	5.08 ± 0.30	5.8	5.06 ± 0.30	5.9
	40	38.6 ± 1.8	4.7	42.5 ± 2.1	5.0	44.0 ± 1.6	3.7	36.3 ± 1.9	3.5
(−)-*R*-DMR	1	0.91 ± 0.079	8.6	1.08 ± 0.12	10.8	1.02 ± 0.088	9.1	1.03 ± 0.078	8.2
	5	4.98 ± 0.25	5.1	4.63 ± 0.23	4.9	5.33 ± 0.29	5.5	4.86 ± 0.26	5.4
	40	39.20 ± 2.1	5.2	37.26 ± 1.8	4.7	40.55 ± 2.6	6.3	39.00 ± 2.0	5.0
(+)-*S*-DMR	1	1.03 ± 0.12	11.1	1.07 ± 0.12	11.6	1.00 ± 0.070	6.8	1.03 ± 0.12	11.7
	5	4.65 ± 0.28	6.0	4.52 ± 0.26	5.7	5.40 ± 0.34	6.2	4.98 ± 0.25	4.9
	40	39.20 ± 2.1	5.2	39.98 ± 2.3	5.6	36.51 ± 1.4	3.9	40.61 ± 1.5	3.6
(−)-*R*-8-OHM	1	0.91 ± 0.081	8.6	1.02 ± 0.11	10.5	1.13 ± 0.10	9.2	0.95 ± 0.09	9.4
	5	5.12 ± 0.22	4.3	5.07 ± 0.18	3.6	4.99 ± 0.25	4.9	5.31 ± 0.36	6.8
	40	39.55 ± 0.93	2.4	40.4 ± 2.0	4.8	42.9 ± 1.8	4.1	40.0 ± 2.0	4.9
(+)-*S*-8-OHM	1	0.97 ± 0.11	10.9	1.01 ± 0.09	8.5	1.02 ± 0.12	12.3	0.98 ± 0.11	10.8
	5	5.10 ± 0.20	3.9	5.06 ± 0.19	3.7	5.04 ± 0.27	5.3	4.93 ± 0.34	6.8
	40	40.12 ± 2.0	5.0	40.71 ± 1.3	3.2	42.55 ± 1.8	4.1	38.72 ± 1.8	4.7

^a^ After three freeze–thaw cycles.; ^b^ Stored at −20 °C; ^c^ Exposed at ambient temperature (25 °C) for 8 h.; ^d^ Kept at ambient temperature (25 °C) for 8 h.

### 2.3. Application to Pharmacokinetic Studies

**Figure 6:**
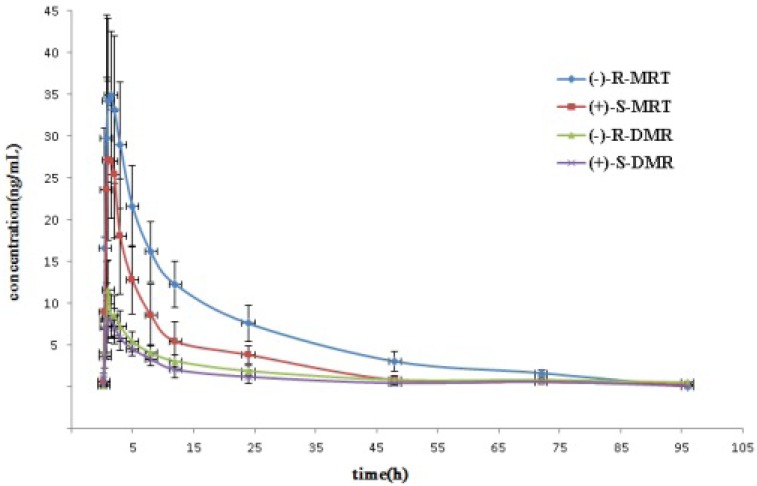
Mean plasma concentration–time profiles for MRT and DMR enantiomers in 12 healthy male volunteers following oral administration of *rac*-MRT 30 mg.

8-OHM was only detected in plasma at 1 h in two volunteers following oral administration of MRT. The majority of plasma samples had no detectable levels of 8-OHM. The reason could be that conjugated 8-OHM was predominantly found in plasma and hydrolysis reaction should be implemented for the assay of 8-OHM in plasma [12,14,20]. Figure 6 represents mean plasma concentration profile of MRT, and DMR enantiomers *versus* time in these volunteers. The maximum concentration of (−)-*R*- and (+)-*S*- MRT were found at approximately 1.5 h after the administration of *rac*-MRT. The concentration-time courses of the individual enantiomers of MRT in plasma were distinctly different. Although the plasma concentration of (+)-*S*-DMR was similar to that of (−)-*R*-DMR at each time point, the *S*/*R*-ratios of DMR were < 1 in all human subjects. The mean AUC_0__−__∞_ values of (−)-*R*-enantiomers were significant higher (*p* < 0.05) than that of (+)-*S*-enantiomers for both MRT and DMR by an independent-measures t-test. Pharmacokinetic parameters estimated by the non-compartmental approach are listed in Table 6. These values were in agreement with previously published data [12,19].

**Table 6:** Pharmacokinetic parameters of the enantiomers of MRT and DMR after oral administration of *rac*-MRT 30 mg to 12 healthy volunteers.

Enantiomer	C_max_ (ng/mL)	T_max_ (h)	t_1/2_(h)	MRT * (h)	AUC_0-96_ (ng h/mL)	AUC_0−∞_ (ng h/mL)
(−)-*R*-MRT	41.81 ± 4.1	1.42 ± 0.66	23.22 ± 4.9	27.37 ± 5.5	570.94 ± 98	613.84 ± 103
(+)-*S*-MRT	34.15 ± 5.9	1.30 ± 0.71	15.54 ± 4.4	17.43 ± 4.0	276.94 ± 76	284.14 ± 74
(−)-*R*-DMR	12.73 ± 2.5	1.32 ± 0.54	19.80 ± 4.3	22.55 ± 5.7	133.75 ± 36	149.83 ± 39
(+)-*S*-DMR	10.02 ± 1.8	1.29 ± 0.73	11.45 ± 3.3	17.90 ± 4.5	96.10 ± 34	109.84 ± 36

* MRT, mean residence time, represents the average time the drug molecule stays in the body.

## 3. Experimental

### 3.1. Chemicals and Reagents

Racemic MRT (98.0% purity) (Figure 1) was purchased from Sigma-Aldrich (St. Louis, MO, USA). Racemic DMR, 8-OHM and N-O-MRT (Figure 1) were supplied by Toronto Research Chemicals Inc (North York, ON, Canada). Olanzapine (99.0% purity, internal standard, Figure 1) was obtained from National Institute for the Control of Pharmaceutical and Biological Product (Beijing, China). CM-β-CD sodium salt (degree of substitution, DS~3.5) was purchased from Cyclodextrin Research & Development Laboratory (Buchs, Switzerland). Sodium dihydrogenphosphate and sodium tetraborate were acquired from China Medicine (Group) Shanghai Chemical Reagent Corporation (Shanghai, China). Phosphoric acid was from Sigma (St. Louis, MO, USA). ACN and methyl *tert*-butyl ether were from J.T. Baker (Phillipsburg, NJ, USA). All chemicals were analytical grade or better. Human control plasma (sodium heparin as an anticoagulant) was obtained from Shanghai Shuguang Hospital (Shanghai, China). Deionized water (18.2 MΩ cm) was obtained by using a Milli-Q system (Millipore, Milford, MA, USA).

### 3.2. Apparatus and Capillary Electrophoretic Conditions

All assays were performed on a CE system consisted of a Beckman P/ACE MDQ instrument (Beckman Coulter, Brea, CA, USA) equipped with a photodiode array detection detector (PDA) and P/ACE System MDQ Software. Data collection, processing, and analysis were performed using system 32-Karat software (Beckman). An uncoated fused-silica capillary (Yongnian Optical Fiber Factory, Hebei, China) of 75 μM i.d., 40.2 cm in total length, and 30.2 cm in effective length was used. Detection was performed at 200 nm, where the samples had the maximum absorption. 

The BGE solution used in this study was composed of 6.25 mM borate–25 mM phosphate (pH 2.8) and 5.5 mg/mL CM-β-CD. It was prepared by accurately weighing 0.39 g phosphate and 0.24 g borate and making up to 100 mL with deionized water. Then 0.55 g CM-β-CD was weighed and added into the borate–phosphate buffer. After that, buffer pH was adjusted to 2.8 with 10% phosphoric acid. The BGE were prepared freshly every day and filtered through a 0.45 μM hydrophilic cellulose membrane filter and degassed by sonication prior to use. The capillary was thermostated at 20 °C. The samples were introduced by electrokinetic injection (7.5 KV for 20 s) and run with the applying optimal voltage of 16 kV with the current of about 80 μA. New uncoated fused-silica capillary was conditioned by flushing with 0.1 mol/L NaOH for 30 min, and then rinsed with 0.1 mol/L HCl and water, respectively for 10 min. Prior to each analysis, the capillary column was rinsed with 0.01 mol/L NaOH for 3 min, and then water, followed by the BGE, each for 2 min between the runs.

### 3.3. Preparation of Stock Solutions, Calibration Samples and Quality Control Samples

Standard stock solution (1 mg/mL) of *rac*- MRT, DMR, 8-OHM, N-O-MRT and the IS were prepared in methanol, respectively. Working standard solutions (rang from 10 to 1000 ng/mL) were prepared daily by diluting suitable aliquots of the stock solution to desired concentration with deionized water. The IS solution was prepared in deionized water at the concentration of 200 ng/mL. The standard solutions were stored in brown glass vial to protect from light at 4 °C.

Calibration samples were obtained by diluting standard working solutions (20 μL) with drug-free human control plasma (180 μL), to span a calibration standard range of 1–100 ng/mL for *rac-*MRT, *rac*-DMR and *rac*-8-OHM. QC samples (2, 10, 80 ng/mL) for *rac*-MRT, *rac*-DMR and *rac*-8-OHM were independently prepared by spiking appropriate amount of the working standard solution in drug-free human control plasma.

### 3.4. Preparation of Human Plasma Sample

Plasma samples were pretreated using LLE in 96-well plates format. An electronic 96 channel handheld pipettor (INTEGRA Biosciences AG, CH-7205 Zizers, Switzerland) was used for liquid transfer steps. Two hundred μL of subject plasma samples, calibration samples and QC samples were spiked into a 2 mL deep 96-well plate, respectively. Aliquots of 20 μL of 200 ng/mL IS solution were added to each well except for the well designated for the double blank plasma. Then 20 μL 1 mol/L NaOH were added to each well of the plate, and the plate was vortex mixed for 30 s. After adding 1.0 mL of methyl *tert*-butyl ether, the plate was covered with a mat, vortex mixed for 5 min, and centrifuged for 10 min at 3500 rpm. Next the mats were carefully removed and 0.8 mL of the supernatant organic layer was transferred from the original sample plates into the respective positions of new 2.0 mL deep 96-well plates. The plates were then placed into a self-constructed 96-well plate evaporator, and the organic extracts were evaporated to dryness with a nitrogen flow at 35 °C. All dry residues were reconstituted by addition of 50 μL ACN–water (80:20, v/v). Finally, the plates were vortex mixed for 5 min, then centrifuged for 10 min at 3500 rpm, and the supernatant was transferred to the autosampler for injection onto the CE system.

### 3.5. Clinical Pharmacokinetic Study

The study protocol was formally accepted by China Food and Drug Administration and approved by a local ethics committee. Informed consent was obtained from all the subjects after explaining the aim and risks of the study. Twelve healthy Chinese male subjects with mean age 30 ± 1.5 years and average weight 64.3 ± 6.5 kg were enrolled into the study. The volunteers received a single oral dose of 30 mg racemic MRT tablets (Remeron^®^, N.V. Organon, 30 mg/ tablet) around 8 a.m., with 200 mL of water after an overnight fasting. Blood samples (1 mL) were collected into heparinized centrifuge tubes at pre-dose (0 h) and at the time of 0.25, 0.5, 0.75, 1, 1.5, 2, 3, 5, 8, 12, 24, 48, 72 and 96 h after administration. Plasma was separated by centrifugation (4,000 *×g*) for 10 min at 4 °C and frozen at −80 °C until analysis.

## 4. Conclusion

An easy, cost effective, rapid and highly sensitive ACN-FASS CZE method has been validated and proposed for the simultaneous *in vitro* enantioselective analysis of MRT and its three metabolites for the first time, which was recommended to be adopted for analyzing pharmacokinetic profiles of MRT in human subjects. CM-β-CD was selected as chiral additive and the optimization procedure demonstrated that electrokinetic injection in conjunction with dissolving the sample in 80% ACN yields better stacking and provided a sensitivity enhancement of about 50–60 folds. After LLE in 96-well plates format, the enantiomers in plasma samples as low as 0.5 ng/mL for MRT, DMR and 8-OHM were able to be separated and detected by CZE with the aid of ACN-FASS within 7 min. The developed and validated method was applied to a stereoselective pharmacokinetics study of MRT and its metabolites in healthy volunteers after oral administration of MRT.
